# Editorial: Tackling the presence of pathogens in water and wastewater

**DOI:** 10.3389/fpubh.2022.1022714

**Published:** 2022-11-16

**Authors:** Maria Tereza Pepe Razzolini, Maria Inês Zanoli Sato, Susana Inés Segura-Muñoz, Raphael Correa Medeiros

**Affiliations:** ^1^Department of Environmental Health, School of Public Health of the University of São Paulo, São Paulo, Brazil; ^2^Department of Environmental Analysis, CETESB—São Paulo State Environmental Company, São Paulo, Brazil; ^3^Laboratory of Ecotoxicology and Environmental Parasitology, Ribeirão Preto College of Nursing, University of São Paulo, Ribeirão Preto, Brazil; ^4^Department of Engineering and Environmental Technology, Federal University of Santa Maria, Frederico Westphalen Campus, Santa Maria, Brazil

**Keywords:** drinking water, water quality, wastewater quality and regulation, water for reuse: quality and challenges, management tools for water resource, wastewater-based epidemiology

The United Nations recognizes water safety, including unhampered access to clean drinking water, as an issue of global importance and declared it a major goal of sustainable development (1). In this context, the Research Topic “*Tackling the presence of pathogens in water and wastewater*,” published in Frontiers, combines articles by research groups dedicated to the study of pathogens relevant to human health in natural or man-made water bodies.

While monitoring and assessment of pathogens in water or wastewater—including, among other taxonomic groups, bacteria, viruses, and proto- or metazoans—are certainly well-established and long-standing Research Topics, this edition makes an attempt to highlight the manifold ways by which these lines of research contribute to the One Health Concept. Well-recognized by the WHO, the One Health Concept has become an important conceptual tool and major pillar of applied environmental sciences. It considers multiple factors determining, interfering with, or even masking the risks of pathogen uptake and transmission, as well as disease outbreak, considering the complex network of interdependencies of environmental factors and those related to human behavior.

The first article by Daer et al. highlights implications for public health of using disinfectants commonly used to treat water, especially drinking water, as this treatment can favor the evolution of resistant pathogen strains. The authors investigated the emergence of resistance to monochloramine and ferrate disinfection in *Escherichia coli* using an experimental approach and demonstrated a potential risk for the development of microbial resistance under a recurrent disinfection regime. An important question emerges from this study, namely, how shortened wastewater treatment cycles, along with the respective disinfection treatment(s), might amplify the emergence of resistant bacterial strains.

The second article by Nogueira et al. represents a pioneer study that assessed viral diversity (i.e., the virome) in the Amazonian Lake Bolonha, which provides drinking water to the local community. The authors sought to quantify the taxonomic diversity of DNA viruses, as well as that of bacterio- and cyanophages, given that phages can transduce antibiotic resistance genes to their hosts. To this end, Nogueira et al. reanalyzed metagenomic sequencing data generated for previous studies. The results suggest a diverse viral community and established phage-regulated dynamic of resident bacterial and cyanobacterial communities. Studying viromes and the spread of antibiotic resistance genes through phages are certainly exciting new venues for the monitoring and assessment of water quality in sources of drinking water.

Next, Senecal et al. shed light on the risk imposed by pathogens contained in human urine for wastewater management. The authors tested methods to inactivate the infectious stages of a metazoan gastrointestinal parasite (*Ascaris suum*) and two viruses (coliphages MS2 and ΦX174). A general observation from this study was that the metazoan parasite appears to be more difficult to inactivate than the two viruses, and so wastewater treatment should prioritize combating gastrointestinal parasites at least at stages when local parasite prevalence is high.

Belgasmi et al. propose a novel approach for detecting polioviruses and other enteroviruses in residual water leaving water treatment plants. Developed by CDC-Atlanta, it has been termed CAFÉ (Concentration and Filtration Elution) and utilizes existing filtration techniques that are suitable for a vast array of sewage or residual waters. The comparison between CAFÉ and the standard two-phase separation currently used by laboratories supported by WHO showed that CAFÉ is a robust, sensitive, and cost-effective method for isolating enteroviruses from residual waters and is easy to apply from the perspective of practicality. Designing similar approaches to detect other pathogens certainly represents a promising and welcome outlook into the future.

Finally, Shiga toxin-producing *E. coli* (STEC) can lead to disease outbreaks especially when food becomes contaminated during production. Maguire et al. examined water samples using Oxford Nanopore rapid sequencing kits with the aim of assessing microbiomes, focusing on the detection and characterization of STEC. Poor nanopore sequencing results led the authors to prepare a genomic library using a ligation kit and including “clean-up” steps; nevertheless, despite 100 reads from each site (0.02% of total reads) being identified as *E. coli*, STEC were not detected. By contrast, when standard microbiological techniques were applied, STEC were detected at three study sites. The authors suggest that the results obtained from rapid sequencing could be improved when applied in combination with a ligation kit to reduce contamination effects.

We really hope that our readers find in this Research Topic valuable references for this challenging field of research and, also, find inspiration to keep being engaged in environmental microbiology science.

## Author contributions

All authors listed have made a substantial, direct, and intellectual contribution to the work and approved it for publication.

## Conflict of interest

The authors declare that the research was conducted in the absence of any commercial or financial relationships that could be construed as a potential conflict of interest.

## Publisher's note

All claims expressed in this article are solely those of the authors and do not necessarily represent those of their affiliated organizations, or those of the publisher, the editors and the reviewers. Any product that may be evaluated in this article, or claim that may be made by its manufacturer, is not guaranteed or endorsed by the publisher.

## References

[1] WHO/UNICEF. Report Progress on Household Drinking Water, Sanitation and Hygiene 2000-2020. Monitoring Programme for Water Supply, Sanitation and Hygiene (JMP). Geneva (2021).

